# Natural autoantibodies and their functional therapeutic roles in intravenous immunoglobulin

**DOI:** 10.3389/fragi.2025.1682457

**Published:** 2025-10-01

**Authors:** Maria Giovanna Danieli, Ilaria Claudi, Elena Buti, Luca Gammeri, Sebastiano Gangemi, Yehuda Julyus Shoenfeld

**Affiliations:** ^1^ Postgraduate School in Allergy and Clinical Immunology, Dipartimento di Scienze Cliniche e Molecolari, Università Politecnica delle Marche, Ancona, Italy; ^2^ SOS Immunologia delle Malattie Rare e dei Trapianti, SOD Clinica Medica, Dipartimento di Medicina Interna, Azienda Ospedaliero Universitaria delle Marche, Ancona, Italy; ^3^ School of Allergy and Clinical Immunology, University of Messina, Messina, Italy; ^4^ Operative Unit of Allergy and Clinical Immunology, Department of Clinical and Experimental Medicine, University of Messina, Messina, Italy; ^5^ Reichman University, Herzelia, Israel; ^6^ Zabludowicz Center for Autoimmune Diseases, Sheba Medical Center, Ramat Gan, Israel

**Keywords:** Alzheimer’s diseases, autoimmunity, connective tissue diseases, epigenetic, intravenous immunoglobulin, natural autoantibodies, Parkinson’s disease, small fiber neuropathy

## Abstract

Natural autoantibodies (NAbs) are a key component of the immune system, produced mainly by B-1 cells without prior antigenic stimulation. These antibodies exhibit broad reactivity toward both self and non-self antigens and contribute to immune homeostasis by clearing apoptotic cells and cellular debris, modulating immune responses, preventing autoimmune reactions, and promoting tissue repair. NAbs are present in intravenous immunoglobulin (IVIg) preparations, where they play an important role in the therapeutic effects observed in autoimmune, inflammatory, and neurodegenerative diseases. Importantly, NAbs of the IgG class contained in commercial IVIg originate from large-scale pooling of sera from thousands of healthy donors, and are recovered after multiple enrichment and purification steps during the manufacturing process. This review provides a comprehensive overview of the physiological functions of NAbs and their involvement in the mechanisms of action of IVIg. The review particularly focuses on anti-idiotypic antibodies within IVIg, which can neutralize pathogenic autoantibodies in diseases such as systemic lupus erythematosus, antiphospholipid syndrome, and pemphigus vulgaris. In the neurological field, NAbs in IVIg have been shown to target misfolded proteins such as amyloid-beta and alpha-synuclein, reduce neuroinflammation, and support neuronal survival, with promising results in Alzheimer’s disease, Parkinson’s disease, autoimmune encephalitis, and small fiber neuropathy. Similarly, in dermatological and systemic autoimmune diseases, NAbs contribute to immune regulation and the neutralization of tissue-damaging autoantibodies. Enhancing the therapeutic potential of IVIg through selective enrichment of beneficial NAb subsets could represent a promising direction for future research aimed at improving outcomes in a wide range of immune-mediated diseases.

## Highlights

• Natural autoantibodies (NAbs) contribute to immune homeostasis, act as a first line of defence, and provide protection against various pathologies;
• Epigenetic processes, such as DNA methylation and histone acetylation, critically influence B-cell maturation and the production of NAbs, highlighting a key regulatory mechanism;
• NAbs possess a broad range of physiological functions, including clearing cellular debris, modulating immune responses by affecting T and B cells, and contributing to tissue repair and protection against autoimmunity;
• Intravenous Immunoglobulin (IVIg) therapy harnesses also the therapeutic potential of NAbs, including anti-idiotypic antibodies, to neutralize pathogenic autoantibodies and modulate immune dysregulation in various diseases;
• NAbs in IVIg offer significant therapeutic benefits in neurodegenerative diseases like Alzheimer’s and Parkinson’s by targeting protein aggregates and neuroinflammation, as well as in various autoimmune and inflammatory conditions;
• In the future, using IVIg formulations enriched with specific NAbs or preparations based on selected NAbs could help create personalized therapeutic approaches for each disease.

## 1 Introduction

Natural autoantibodies (NAbs), a fundamental component of the immune system, are immunoglobulin glycoproteins produced especially by a subtype of B lymphocytes, B-1 cells, in the absence of specific antigenic stimulation (Kaveri and Bayry, 2017; Lobo, 2017). These antibodies, like the autoantibodies that emerge in various immunopathological conditions, utilize the same genetic elements as antibodies targeting environmental antigens. Avrameas suggests that NAbs, by interacting with the vast array of self-components present in an organism, establish a broad and dynamic network that contributes to the body’s overall homeostasis (Avrameas, 1991). NAbs, often belonging to the IgM isotype at low affinity (IgM-NAbs), but also IgG and IgA in smaller percentages, are encoded by genes present in the germline in the variable region and are distinguished by a broad reactivity, recognizing both self and non-self antigens (Kaveri and Bayry, 2017; Lobo, 2017; Kaveri, 2012; Grönwall and Silverman, 2014). They act as a first line of defence against invading pathogens and play a crucial role in maintaining immune homeostasis (Ochsenbein et al., 1999; Miescher and Käsermann, 2013; Avrameas et al., 2018). NAbs maintain tissue homeostasis and play a protective role in bacterial and viral infections, chronic inflammatory diseases, cardiovascular disorders, and neurodegenerative conditions. These biological activities of NAbs can also be observed following intravenous immunoglobulin (IVIg) administration and may contribute at various levels to their disease-protective effects (Avrameas and Selmi, 2013). NAbs levels and their repertoire can vary between healthy individuals and under pathological conditions, influencing the inflammatory response (Grönwall and Silverman, 2014). Table 1 shows NAbs and their targets detected in selected diseases.

**TABLE 1:** Targets of natural autoantibodies detected in selected diseases.

Disease	Target of NAbs
Alzheimer’s disease	Amyloid-beta
	Tau
Parkinson’s disease	α-synuclein
Rheumatoid arthritis	Glycosaminoglycans
	T-cell receptor
Systemic lupus erythematosus	Phosphorylcholine
Immune thrombocytopenic purpura	Platelet glycoproteins
Pemphigus vulgaris	Desmoglein-1 and desmoglein-3

**FIGURE 1:**
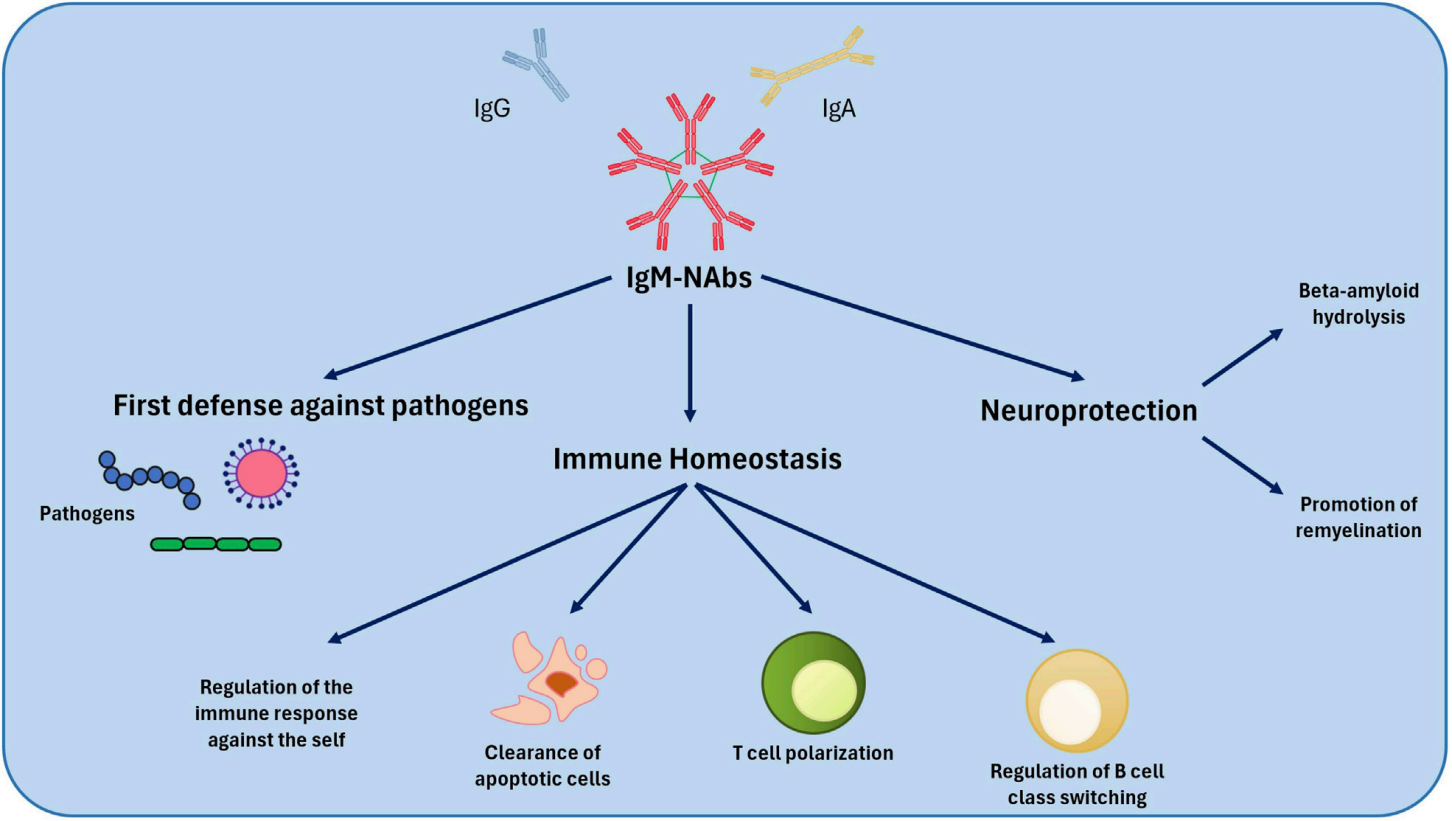
Mechanism of action of NAbs. NAbs play a defensive role against pathogenic microorganisms and regulate immune system homeostasis. Among their effects, NAbs also play a neuroprotective role. NAbs, Natural Autoantibodies.

**FIGURE 2:**
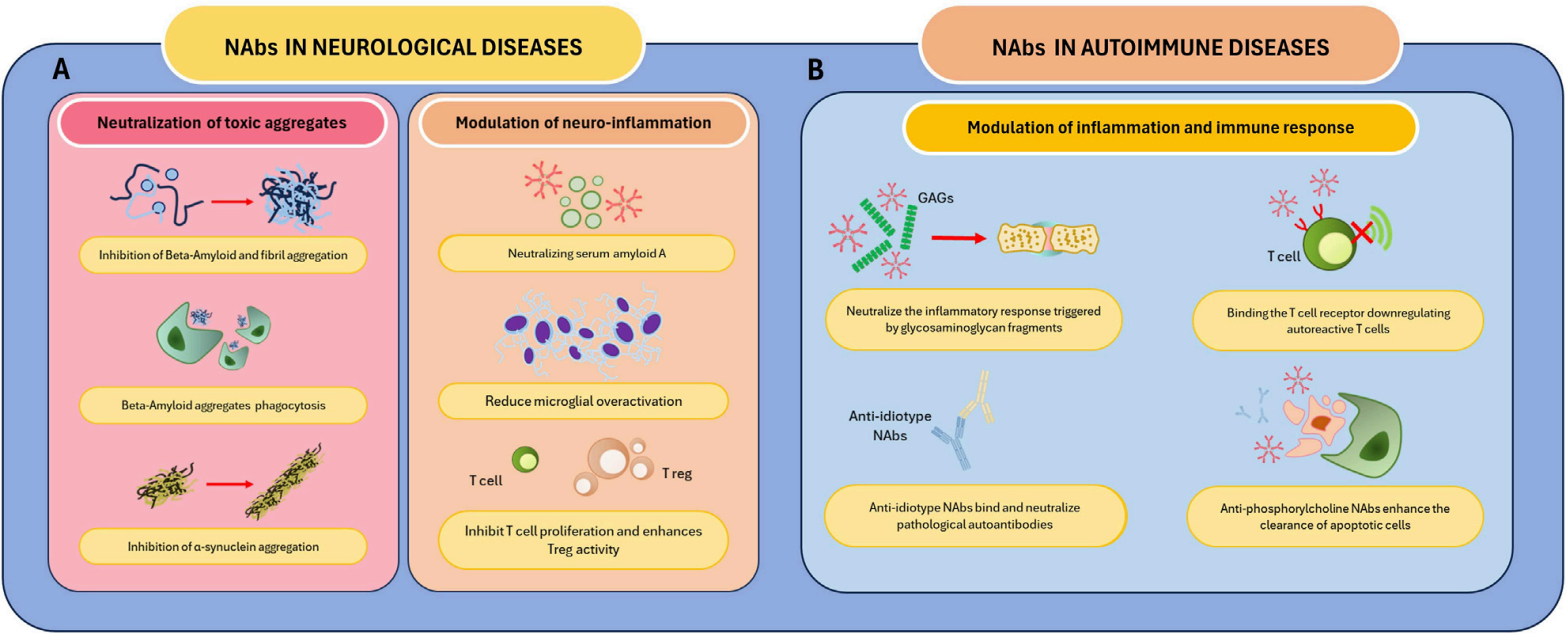
Mechanism of action of Nabs in selected diseases. NAbs play a role in the treatment of neurological and autoimmune diseases. **(A)** NAbs mediate the neutralization and clearance of neurotoxic substances responsible for neurological diseases such as PD and AD. These autoantibodies modulate neuroinflammation by neutralizing SSA, regulating T cell activity, and reducing microglial activation. **(B)** NAbs neutralize GAGs and anti-idiotype antibodies, reducing the inflammatory response and, in the latter case, neutralizing pathological autoantibodies. Furthermore, these autoantibodies downregulate the activity of autoreactive T cells by binding the TCR and enhance the clearance of apoptotic cells. Abbreviations: AD, Alzheimer’s disease; GAGs, Glycosaminoglycans; PD, Parkinson’s disease; SSA, Serum amyloid A; TCR, T cell receptor.

NAbs can be measured in patient serum using immunoenzymatic methods such as ELISA. However, measuring these antibodies is not a routine test, so not all laboratories are equipped to search for NAbs. In most cases, these autoantibodies are measured for research purposes using kits that can be purchased from specialized supplier companies.

This review aims to provide a comprehensive analysis of the physiological functions and therapeutic applications of NAbs, with a particular focus on their role in IVIg full components. By examining current literature, we will explore how NAbs contribute to immune regulation, neuroprotection, and inflammation control, as well as their potential implications for future clinical applications. Additionally, we will discuss strategies for enhancing the therapeutic efficacy of IVIg through targeted antibody enrichment and personalized treatment approaches.

### 1.1 Functional capacity of natural autoantibodies

The removal of cellular debris and apoptotic cells represents one of the most important physiological functions of NAbs: IgM-NAbs facilitate their elimination through phagocytes. This process is crucial to prevent unwanted inflammatory responses (Lobo, 2017; Fereidan-Esfa et al., 2019). Some studies suggest that IgM-NAbs levels can be influenced by infections, maintaining protective levels and explaining the low incidence of autoimmune diseases in some geographic areas (Lobo, 2017). Some Nabs, particularly IgM, contribute to tissue repair in the central nervous system, favouring remyelination in multiple sclerosis models (Fereidan-Esfa et al., 2019). Moreover, NAbs exert an important immune modulation action, driving the polarization of T cells and the class switch of B cells. This capacity is crucial to regulate the intensity and specificity of the immune response (Fereidan-Esfa et al., 2019). Another function is the protection against autoimmunity: NAbs contribute to the protection of the organism against autoimmune inflammation (Lobo, 2017).

NAbs show their potential function in recognition of oxidized epitopes as in atherosclerosis, modulation of inflammation, by binding to different receptors and inflammatory mediators (Lobo, 2017; Schwartz-Albiez et al., 2009). Several authors reported a possible role in cancer, with NAbs recognizing specific oligosaccharides expressed on the surface of tumour cells, exerting an antitumor function or against vascular endothelial growth factor (anti-VEGF), which can inhibit angiogenesis, a fundamental process for tumour growth (Schwartz-Albiez et al., 2009).

Moreover, some NAbs seem to have catalytic activity, able to hydrolyse peptides such as amyloid-beta, involved in the pathogenesis of Alzheimer’s disease, whereas other cationized forms of NAbs can cross the blood-brain barrier, suggesting a potential therapeutic role in central nervous system disorders (Fereidan-Esfa et al., 2019; Trépanier et al., 2012).

### 1.2 Role of epigenetics in NAbs production

Epigenetics influences the production of NAbs. Several studies have shown that B cells’ maturation, and therefore B1 cells, are strictly linked to specific epigenetic processes (Xiao et al., 2022). For example, Mahaja et al. (2021) have demonstrated, through the analysis of CpG methylation, that the basis of the development of B1a cells is driven by the programmed demethylation of enhancers. These are instead methylated in B2 cells. The same authors noted that the DNMT3a-dependent CpG methylation can control the specific gene expression of B cells, and that the deficiency of this enzyme leads to the selective expansion of B1a cells. da Costa et al. (2020) instead hypothesized that histone acetylation plays a key role in regulating the functions of these cells. In their studies, they demonstrated that the inhibition of the histone deacetylase enzyme helps the migration of B1 cells. In several diseases, the cytokine environment can promote some epigenetic modifications, which can be reflected in the activity of B1 cells. Garaud et al. (2009) have demonstrated how IL-6 in patients with systemic lupus erythematosus (SLE) can regulate, through DNA demethylation processes, the expression of CD5, an important marker of B1 cells.

### 1.3 Therapeutic potential of NAbs

The therapeutic applications of NAbs have gained increasing attention, particularly in the context of IVIg therapy. IVIg, a plasma-derived preparation containing a mixture of antibodies from healthy donors, has demonstrated significant immunomodulatory potential in autoimmune diseases, inflammatory disorders, and neurological conditions (Kaveri, 2012; Schwartz-Albiez et al., 2009). IVIg contains IgG-NAbs directed against variable and constant regions of the human αβ T-cell receptor, CD5, CD4, HLA I molecules, RGD adhesion motif, CCR5, Fas, cytokines, and cytokine receptors. These antibodies are critical for the immunomodulatory effects of IVIg (Vani et al., 2008).

The presence of NAbs within IVIg contributes to its ability to neutralize pathogenic autoantibodies, modulate Fc receptor activity, and regulate complement activation, making it a valuable therapeutic strategy for conditions such as Guillain-Barré syndrome, multiple sclerosis, and rheumatoid arthritis (Seite et al., 2008; Ramos-Medina et al., 2012).

Recent advances in research have highlighted the potential of specific NAbs, including anti-FcεRIα modulating immune responses and providing neuroprotection (Kaveri and Bayry, 2017; Bouhlal et al., 2014). Furthermore, emerging data indicate that optimizing IVIg formulations to enhance NAbs fractions could improve clinical outcomes in inflammatory and neurodegenerative disorders (Fereidan-Esfahani et al., 2019).

Among the NAbs present in IVIg, there are also cell-penetrating antibodies, NAbs capable of entering cells and localizing in the cytoplasm. These cell-penetrating antibodies exert an inhibitory effect on immune cell activation, contributing to the immunoregulatory properties of IVIg (Sali et al., 2015).

Sarrigeorgiou et al. (2024) have shown how NAbs levels are reduced in patients with Common Variable Immunodeficiency compared to healthy subjects. In addition, a direct correlation between the reduced levels of NAbs and specific clinical phenotypes was noticed. The levels of NAbs, of the IgG class, were restored after replacement therapy with IVIg, demonstrating how, in the future, it could be helpful to resort to the administration of specific NAbs to improve the clinical response in specific disease phenotypes.

## 2 Natural autoantibodies in intravenous immunoglobulin

NAbs in IVIg play a key role in immune regulation by maintaining homeostasis, clearing apoptotic cells, and modulating inflammation. Their ability to recognize conserved molecular structures has led to the development of targeted therapies for neurological and autoimmune diseases.

### 2.1 Anti-idiotypic antibodies in IVIg

Anti-idiotypic antibodies (anti-Id) are antibodies that bind to the variable region (idiotype) of other antibodies. This interaction can neutralize the original antibody, regulate immune responses, or activate immune cells. Niels Kaj Jerne’s idiotypic network theory describes the immune system as a self-regulating network where antibodies influence each other (Jerne and Cocteau, 1984). When an antibody (Ab1) binds to an antigen, it presents a unique molecular pattern (idiotype) that can be recognized by another antibody (Ab2), the anti-idiotypic antibody. This creates a feedback loop where antibodies regulate each other’s activity, amplifying or suppressing the immune response as needed. Jerne proposed that this network helps maintain immune balance and prevents autoimmunity by suppressing harmful self-reactive antibodies. Some anti-idiotypic antibodies can even mimic the original antigen’s structure, allowing the immune system to “remember” pathogens without continuous exposure (Jerne and Cocteau, 1984).

A proposed mechanism for the beneficial effects of IVIg therapy involves the activity of anti-idiotypic antibodies against pathogenic autoantibodies. IVIg is widely used for its immunomodulatory properties, and part of its mechanism of action involves the regulation of the idiotypic network. IVIg preparations contain anti-idiotypic antibodies, derived from the pooled plasma of numerous healthy donors. Some of these anti-idiotypic antibodies derive from exposure to antigens, others are NAbs. Anti-idiotypic antibodies are thought to play a key role in the immunoregulatory effects of IVIg in immune-mediated disorders (Blank et al., 2014). Anti-idiotypic antibodies within IVIg, including anti-idiotypic NAbs, can bind to and neutralize idiotypes on pathogenic autoantibodies, thereby modulating immune responses and reducing autoimmune activity (Blank et al., 2014).

The idiotypic network forms the foundation for understanding how IVIg exerts its immunomodulatory effects. Anti-idiotypic antibodies present in IVIg can bind to the idiotypes of pathogenic autoantibodies, leading to their neutralization or to the modulation of the B cells producing them. This ability to interact with the idiotypic network is considered one of the most important mechanisms through which IVIg exerts its beneficial effects in autoimmune diseases. Several studies have provided concrete evidence of the presence and role of specific anti-idiotypic antibodies within IVIg preparations in various autoimmune contexts (Blank et al., 2014).

An important advancement in the therapeutic potential of IVIg comes from the development of specific IVIg fractions (sIVIg) through affinity purification techniques. As Blank et al. (2005) reported, sIVIg can be produced by a three-step process. First, an autoantigen column is prepared for the affinity purification of autoantibodies; second, the purified autoantibodies are used to create a new column composed of the autoantibodies themselves; finally, this column is used to affinity purify anti-autoantibodies (anti-idiotypes) from IVIg, resulting in autoimmune disease-specific IVIg (sIVIg). Studies have shown that sIVIg is more effective than whole IVIg in experimental models of autoimmune diseases, highlighting the potential for enhanced immunomodulatory therapies through targeted anti-idiotypic antibody enrichment (Blank et al., 2014). The beneficial effects of anti-idiotypic antibodies within IVIg are also evident in recurrent pregnancy loss. Konova et al. (2007) observed that serum anti-elastin IgG autoantibodies were significantly higher in patients with recurrent pregnancy loss compared to healthy controls. Interestingly, anti-elastin and anti-anti-elastin idiotypes were identified in all tested IVIg lots. This suggests that the presence of anti-idiotypes targeting anti-elastin NAbs within IVIg could represent an additional mechanism through which IVIg exerts its therapeutic effects in cases of reproductive failure (Konova et al., 2007).

Another example is antiphospholipid syndrome (APS), where IVIg has shown promise through the activity of specific anti-idiotypic antibodies. A specialized fraction of IVIg, known as sIVIg APS, is enriched with anti-idiotypes targeting autoantibodies involved in APS (Blank et al., 2007). sIVIg APS has been shown to significantly reduce human trophoblast invasion *in vitro* and enhance matrix metalloproteinases MMP2 and MMP9 production, which are essential for embryonic implantation and placental development. In APS, autoantibodies can disrupt these processes, leading to pregnancy complications such as fetal loss. The ability of sIVIg APS to restore normal trophoblast function suggests its potential as a therapeutic compound for early fetal loss in APS patients (Blank et al., 2007).

The therapeutic potential of anti-idiotypic antibodies is further highlighted in pemphigus vulgaris (PV), an autoimmune disease characterized by blisters caused by autoantibodies against desmogleins 1 and 3. Mimouni et al. (2010) demonstrated that a pemphigus vulgaris-specific IVIg preparation (PV-sIVIg), enriched with anti-desmoglein anti-idiotypic antibodies, was more effective than standard IVIg in inhibiting anti-desmoglein-induced pemphigus in mice. *In vitro*, PV-sIVIg significantly inhibited the binding of anti-desmogleins 1 and 3 to desmoglein-3, and *in vivo* it prevented blister formation and the deposition of IgG in the intercellular spaces of the epidermis (Mimouni et al., 2010). This suggests that targeting specific idiotypes involved in PV may enhance the therapeutic efficacy of IVIg.

In SLE, the role of anti-idiotypic antibodies within IVIg is particularly well-documented. Shoenfeld et al. (2002) conducted a study on an experimental murine model of SLE, demonstrating that mice treated with anti-double-stranded DNA (anti-dsDNA) anti-idiotypic antibodies purified from IVIg showed a significant reduction in anti-dsDNA antibody levels, improved proteinuria (a marker of kidney damage), and increased survival compared to mice treated with standard IVIg. The improvement in renal function and the altered patterns of IgG deposition in the kidneys underscore the potential of concentrated anti-idiotypic antibodies as a more targeted and effective therapy for SLE (Shoenfeld et al., 2002).

In atherosclerosis, where oxidized low-density lipoprotein (oxLDL) plays a key pathogenic role, IVIg also demonstrates immunomodulatory effects through anti-idiotypic antibodies. Wu et al. (2003) showed that different commercial IVIg preparations have varying degrees of reactivity towards oxLDL. Through absorption assays, they identified the presence of anti-idiotypes NAbs against anti-oxLDL antibodies in IVIg preparations, suggesting that IVIg could modulate atherosclerosis by interacting with autoantibodies directed against oxLDL. This supports the concept that the modulation of autoantibodies through anti-idiotypic interactions is a key mechanism by which IVIg influences immune responses in cardiovascular diseases.

The ability to isolate and concentrate specific anti-idiotypic antibodies and NAbs from IVIg opens the door to more targeted therapies. While IVIg exerts its immunomodulatory effects through different mechanisms, such as Fc receptor blockade, cytokine network modulation, and increased IgG catabolism, the enhanced efficacy observed with concentrated anti-idiotypes suggests that manipulating the idiotypic network could offer a more specific and efficient therapeutic approach for autoimmune diseases.

### 2.2 Anti-phosphorylcholine antibodies in IVIg

Anti-phosphorylcholine (anti-PC) antibodies, particularly those of the IgM class, are believed to be protective against atherosclerosis and are associated with positive outcomes in hypertensive patients (Wu et al., 2003; Ajeganova et al., 2021). Research suggests that low levels of anti-PC antibodies are linked to the rapid progression of carotid intima-media thickness (C-IMT), a marker for subclinical atherosclerosis, and an increased risk of cardiovascular issues in men (Gigante et al., 2014). Conversely, elevated levels of IgM anti-PC antibodies have been associated with a decreased risk of cardiovascular events in individuals with RA, especially among younger patients and those at high cardiovascular risk (Ajeganova et al., 2021).

In SLE, reduced levels of anti-PC are observed compared to healthy controls. However, when extracted from IVIg, these NAbs exhibit anti-inflammatory properties (Su et al., 2008; Zandman-Goddard et al., 2009). Passive administration of anti-PC antibodies in animal models has demonstrated the ability to inhibit the development of atherosclerosis (Su et al., 2008). Phosphorylcholine is found on apoptotic cells and oxidized LDL (oxLDL), and anti-PC IgG-NAbs may facilitate the removal of these pro-inflammatory agents and prevent the formation of foam cells, a critical process in atherosclerosis (Wu et al., 2003).

IVIg contains NAbs, including anti-PC NAbs, that can modulate immune responses and protect against the progression of various diseases. For example, anti-PC NAbs has shown IVIg’s protective role in reducing lupus inflammation (Su et al., 2008).

In summary, anti-PC antibodies appear to play a complex and potentially protective role in various autoimmune and cardiovascular diseases. Their presence in IVIg contributes to the immunomodulatory effects of this therapy.

### 2.3 Anti-siglec antibodies in IVIg

NAbs targeting sialic acid-binding immunoglobulin-like lectins (Siglecs) represent an emerging area of interest in the therapeutic use of IVIg. Siglecs are transmembrane receptors predominantly expressed on immune cells, where they modulate cellular activation and inflammatory responses. Among these, Siglec-9 plays a critical role in negatively regulating neutrophil activity and promoting anti-inflammatory signalling pathways. Anti-Siglecs antibodies can be either natural (NAbs targeting Siglecs) or induced autoantibodies that arise in response to immunization or disease (Schaub et al., 2011).

Schaub et al. (2011) demonstrated that dimeric IVIg preparations contain NAbs targeting Siglecs and their corresponding anti-idiotypes. This dual presence suggests a dynamic immunoregulatory mechanism in which the balance between agonistic and antagonistic effects could influence the therapeutic activity of IVIg. The study proposed that the idiotype-anti-idiotype interactions may contribute to the suppression of excessive inflammatory responses, offering a potential explanation for some of the anti-inflammatory properties observed in clinical applications of IVIg (Schaub et al., 2011).

Von Gunten and Simon (2008) explored the implications of NAbs targeting Siglecs for immune modulation. They hypothesized that these antibodies act as endogenous regulators of Siglec-mediated signalling pathways, contributing to immune tolerance and the resolution of inflammation. The ability of IVIg to modulate Siglec activity through these NAbs could provide therapeutic benefit in inflammatory conditions or immune dysregulation (Von Gunten and Simon, 2008). The therapeutic potential of NAbs targeting Siglecs has been explored in various diseases characterized by chronic inflammation and autoimmunity, where their role in regulating neuroinflammation is of particular interest (Von Gunten and Simon, 2008). In systemic autoimmune diseases such as rheumatoid arthritis, NAbs targeting Siglecs may help suppress inflammatory responses by modulating neutrophil activation and promoting immune tolerance (Schaub et al., 2011). Furthermore, other inflammatory diseases, such as Eosinophilic granulomatosis with polyangiitis and hypereosinophilic syndrome, could benefit from IVIg-mediated Siglec pathway modulation due to its anti-inflammatory effects on endothelial and immune cells (Von Gunten and Simon, 2008). Future therapeutic strategies might involve the enrichment or selective targeting of these antibodies to enhance the efficacy of IVIg.

### 2.4 Anti-IgE antibodies in IVIg

IVIg preparations also contain anti-IgE IgG autoantibodies (anti-IgE NAbs). These represent approximately 0.3%–0.5% of the total IgG in IVIg (Galeotti et al., 2020). Anti-IgE NAbs are found in healthy individuals, and in asthmatic patients. These autoantibodies are involved in pro-inflammatory and regulatory processes (Chan et al., 2014). The study by Chan et al. has demonstrated that these NAbs exert a dual effect: some are inhibitory, while others activate basophils (Chan et al., 2014). They act differently from omalizumab, an exogenous monoclonal anti-IgE IgG antibody which has the potential to reduce the frequency and severity of asthma and other allergic conditions. Omalizumab binds only free IgE, preventing its interaction with basophils. NAbs, however, can bind both free IgE and IgE bound to its high-affinity receptor FcεRI on the basophils’ surface (Chan et al., 2014). This dual behaviour can explain why some atopic individuals do not have clinical symptoms or why omalizumab therapy has variable efficacy among patients with severe asthma.

IVIg exerts anti-inflammatory effects through multiple mechanisms. One proposed mechanism underlying its efficacy in autoimmune disease is the stimulation of IL-4 production by basophil, which enhances IL-33 activity on SIGN-R1–positive innate cells, leading to an anti-inflammatory effect (Galeotti et al., 2019). Galeotti et al. (2019) demonstrated that IVIg can directly stimulate basophils, resulting in increased expression of CD69, a cell activation marker, thus secreting principal cytokines such as IL-4, IL-6, and IL-8. Upregulation of CD69 on basophils was also observed in IVIg-treated patients with myopathy, confirming the *in vitro* findings. IVIg induces IL-4 production by interacting with IgE already bound on the basophil surface. These results outline a mechanism by which IVIg can promote a Th2-type immune response by the direct interaction of IgG with basophils. In the 2020 work of Galeotti et al. (2020), the induction of IL-4 in basophils was hypothesized to suppress Th1 and Th17 effector cells, upregulate the inhibitory receptor FcγRIIB on phagocytic cells, and reduce the overall inflammatory response. Accordingly, distinct NAbs appear to diversify the effects of IVIg on granulocytes (Galeotti et al., 2020).

## 3 Therapeutic applications of NAbs in IVIg in neurological diseases

IVIg has shown significant immunomodulatory and neuroprotective effects in neurological diseases. These effects are primarily driven by NAbs, which target key pathological mechanisms such as protein aggregation, neuroinflammation, and autoimmunity. Recent studies have shown the mechanisms through which IVIg mediates these benefits, providing promising avenues for its use in neurodegenerative and autoimmune disorders (Table 2).

**TABLE 2:** Natural autoantibodies present in intravenous immunoglobulin and their role in neurological diseases.

Publications	Type of study	Summary
Sim et al. (2020)	Review in AD and PD	Diagnostic and therapeutic role of NAbs in IVIg in AD and PD.
Magga et al. (2010)	Mouse model study in AD	NAbs to amyloid-beta in IVIg protect against amyloid-beta toxicity through multiple mechanisms, including removal of natively formed brain amyloid-beta deposits
Wang et al. (2016)	Mouse model study in AD	NAbs targeting Aβ oligomers provided superior therapeutic effects in a mouse model of Alzheimer’s disease
Kuret et al. (2018)	*In vitro* study in AD	NAbs in IVIg neutralize serum amyloid A, reducing IL-6 release from peripheral blood mononuclear cells and neuroinflammation
Cao et al. (2020)	Research study in AD	Investigated the affinity of IVIG-derived NAbs for different Aβ aggregates
Szabo et al. (2008)	Review in AD	Explored the role of NAbs and NAbs in IVIg targeting amyloid-beta peptide, highlighting their diagnostic and therapeutic potential
Balakrishnan et al. (2010)	Comparative analysis in AD	Comparison of different IVIg formulations, showing higher efficacy for products enriched in NAbs against oligomeric amyloid beta
Bach and Dodel (2012)	Review in AD	Revision of the importance of NAbs diversity in modulating central and peripheral immune responses in AD.
Loeffler (2023)	Review in AD	Reviewed mechanisms of antibody-mediated clearance of amyloid-beta, comparing natural and monoclonal antibodies, and their downstream effects in AD.
Huang et al. (2019)	Mouse model study in PD	NAbs against alpha-synuclein in IVIg improved motor and memory deficits, reduced aggregation, and mitigated neuroinflammation in Parkinson’s models
Braczynski et al. (2022)	*In vitro* and *in vivo* study in PD	In PD, alpha-synuclein-specific NAbs inhibit alpha-synuclein aggregation and reduce neuronal damage both *in vitro* and *in vivo*
Rabenstein et al. (2019)	Research study in PD	The interaction of NAbs in IVIg against toxic alpha-synuclein fragments has a role in neutralization and alpha-synuclein aggregation prevention in PD.
Albus et al. (2018)	Review in proteinopathies	Therapeutic potential of NAbs against misfolded proteins, including alpha-synuclein, amyloid-beta, and Prion protein in proteinopathies like PD.
Karagianni et al. (2019)	Case report in AE	IVIg effectiveness in AE triggered by infection, by reducing inflammation and preventing neuronal damage
Dimitriadou et al. (2020)	Review on anti-neuronal Nabs in IVIg	The role of anti-neuronal antibodies, including Nabs, in IVIg, neutralizing pathogenic autoantibodies, and modulating inflammation by suppressing complement activation and cytokine release
Yang and Liu (2021)	Review in AE	Review of IVIg efficacy in refractory AE, emphasizing its role in neutralizing circulating autoantibodies and modulating immune pathways
Lee et al. (2022)	Multicenter clinical trial in AE	Confirmed the safety and efficacy of IVIg in treating AE through a multicenter clinical trial
Hill et al. (2023)	Multicentre randomized placebo-controlled trial (Ig-NiTE) in AE	IVIg significantly improved clinical outcomes in pediatric encephalitis, emphasizing timely intervention to reduce long-term sequelae
Connolly and Pestronk (1997)	Research study in polyneuropathies	Natural anti-tubulin autoantibodies as biomarkers for disease activity and response to IVIg in acquired demyelinating polyneuropathies
Menon et al. (2021)	Review in atypical CIDP	Review of treatment approaches for atypical CIDP, emphasizing early IVIg use to prevent irreversible nerve damage
Cornblath et al. (2022)	Randomized clinical trial in CIDP	Comparison of different IVIg dosages in CIDP, confirming dose-dependent efficacy and improved muscle strength and functional outcomes

Abbreviation: Aβ, amyloid-beta; AD, Alzheimer’s disease; AE, autoimmune encephalitis; CIDP, chronic idiopathic demyelinating polyradiculopathy; GBS, Guillain-Barré syndrome; IVIg, Intravenous immunoglobulin; NAbs, natural antibodies; PD, Parkinson’s disease.

IVIg has a therapeutic role in the treatment of neurodegenerative diseases, as they are capable of clearing misfolded and aggregated proteins that are responsible for disorders such as Alzheimer’s disease and PD. The study by Sim et al. (2020) highlighted evidence supporting the role of IVIg in reducing amyloid-beta deposition and attenuating tau pathology in Alzheimer’s models, as well as in mitigating alpha-synuclein-mediated toxicity in PD. These findings underline the dual capacity of IVIg to address both proteinopathies and the associated inflammatory cascades.

Rodriguez et al. (2009) identified human NAbs that, when introduced into animal models of human disease, contributed significantly to remyelination of lesions in central nervous system (CNS) demyelinating disorders, offering neuroprotection and promoting neuronal outgrowth in CNS axonal conditions, or bind to immune dendritic cells to enhance the production of cytotoxic T cells that target and eliminate metastatic tumors. The authors also discussed the therapeutic relevance of polyreactive IgM and IgG NAbs, which play a role in maintaining immune homeostasis and reducing tissue damage caused by chronic inflammation (Rodriguez et al., 2009).

Recent studies highlight the potential of NAbs in IVIg to target neurological disease mechanisms, particularly through NAbs against neurotoxic proteins like alpha-synuclein, tau, and amyloid-beta, with promising diagnostic and therapeutic applications. Kuhn et al. (2018) examined serum titres of naturally occurring autoantibodies against alpha-synuclein and tau across different age groups, examining their natural variation and functional relevance. Their findings suggested that these NAbs, which are present even in healthy individuals, play a role in maintaining protein homeostasis by binding and neutralizing potentially toxic aggregates. Importantly, the study highlighted age-related changes in NAbs levels, with implications for early diagnosis and potential treatment strategies for neurodegenerative disorders, such as Alzheimer’s and PD (Kuhn et al., 2018).

Furthermore, Albus et al. (2018) focused their research on NAbs against amyloid-beta, prion protein, and alpha-synuclein. Their work demonstrated that these NAbs bind to pathological aggregates and interfere with them. This finding underscores their therapeutic potential in limiting the progression of proteinopathies. The study also proposed that IVIg preparations enriched in these specific antibodies could serve as a precision-based therapeutic tool to target disease-specific protein aggregates in conditions like Alzheimer’s, Creutzfeldt-Jakob disease, and PD (Albus et al., 2018).

In 2020, Dimitriadou et al. (2020) provided a comprehensive review of anti-neuronal NAbs present in IVIg preparations, emphasizing their importance in clinical practice. They described how these NAbs mediate immunoregulatory and neuroprotective effects by modulating neuroinflammatory responses and enhancing neuronal survival. Specifically, the study documented NAbS directed against linear epitopes to aquaporin-4 and glutamic acid decarboxylase (GAD). The authors proposed that the therapeutic efficacy of IVIg could be linked to the specific repertoire of anti-neuronal NAbs it contains, suggesting a path toward optimizing IVIg formulations for neurological applications (Dimitriadou et al., 2020).

Together, these findings support the continued exploration of these NAbs for developing targeted therapies in a variety of neurological diseases, with a particular focus on their anti-proteinopathy and immunomodulatory properties.

### 3.1 IVIg in Alzheimer’s disease

Alzheimer’s disease (AD) is a neurodegenerative disorder characterized by amyloid-beta (Aβ) plaques, tau tangles, and chronic neuroinflammation, leading to synaptic dysfunction and cognitive decline. IVIg, containing NAbs against Aβ and tau, has emerged as a promising therapeutic approach for mitigating Alzheimer’s disease pathology (Loeffler, 2023). These NAbs possess unique properties that allow them to target toxic Aβ species, modulate inflammatory responses, and protect against neuronal damage. There are different mechanisms of NAbs involved in Alzheimer’s disease therapy. One of the key aspects of IVIg therapy in Alzheimer’s disease is its ability to target different forms of Aβ aggregates, with a particular focus on the oligomeric forms of Aβ, which are believed to be the most neurotoxic.

NAbs present in IVIg contribute to this function through two mechanisms: facilitating the phagocytosis of Aβ aggregates (Magga et al., 2010; Szabo et al., 2008) and inhibiting their aggregation by preventing the nucleation and elongation of toxic Aβ oligomers and fibrils (Magga et al., 2010; Szabo et al., 2008).

In 2019, Cao et al. (2020) demonstrated that NAbs within IVIg exhibit a strong affinity for oligomeric Aβ compared to monomeric or fibrillar forms. This specificity is crucial, as oligomers are known to interfere with synaptic function and contribute to the early stages of Alzheimer’s disease pathology. The ability of IVIg-derived NAbs to bind these toxic aggregates not only prevents their propagation but also facilitates their clearance from the brain.

Supporting this, Wang et al. (2016) showed that NAbs specifically targeting Aβ oligomers provided superior therapeutic effects in a mouse model of Alzheimer’s disease compared to standard IVIg formulations. The study revealed significant reductions in amyloid burden, improved cognitive performance, and restored synaptic integrity, underscoring the therapeutic potential of these antibodies in halting disease progression.

NAbs in IVIg also modulate the chronic inflammation associated with AD, characterized by microglial overactivation and elevated pro-inflammatory cytokines. Kuret et al. (2018) showed that IVIg-derived NAbs neutralize serum amyloid A (SAA), a key mediator of inflammation, reducing IL-6 release and mitigating neuronal damage. This anti-inflammatory action highlights the broader neuroprotective role of IVIg in Alzheimer’s disease.

The variability in IVIg products and their effectiveness in targeting Aβ species has also been a subject of investigation. Balakrishnan et al. (2010) compared different IVIg formulations and found that their efficacy depended on the concentration and specificity of NAbs against Aβ. Products with higher affinity for oligomeric Aβ showed enhanced binding and clearance, suggesting that the therapeutic benefits of IVIg could be optimized by enriching specific antibody profiles. Similarly, Bach and Dodel (2012) emphasized the importance of NAbs diversity in IVIg, highlighting their role in modulating both central and peripheral immune responses. By inhibiting microglial overactivation and reducing systemic inflammation, these antibodies create a more favorable environment for neuronal survival.

In conclusion, NAbs present in IVIg provide a comprehensive therapeutic approach to Alzheimer’s disease, addressing both amyloid pathology and the inflammatory processes that contribute to neurodegeneration. The evidence from preclinical and clinical studies emphasizes the potential of IVIg as a disease-modifying therapy.

### 3.2 IVIg in Parkinson’s disease

Parkinson’s disease (PD) is a progressive neurodegenerative disorder primarily characterized by the pathological accumulation of α-synuclein aggregates. NAbs have emerged as potential therapeutic tools. Recent studies have highlighted the mechanisms and therapeutic potential of NAbs targeting α-synuclein, focusing on their ability to mitigate α-synuclein pathology (Huang et al., 2019; Braczynski et al., 2022; Rabenstein et al., 2019).

In a mouse model, Huang et al. (2019) demonstrated that NAbs against α-synuclein improved memory and motor deficits by reducing aggregation, clearing toxic fibrils, and mitigating neuroinflammation. These NAbs crossed the blood-brain barrier, highlighting the potential of IVIg as a therapeutic strategy for PD.

Braczynski et al. (2022) supported these results by showing that NAbs targeting α-synuclein inhibited the aggregation process both *in vitro* and *in vivo*. These findings confirmed the capacity of NAbs to bind α-synuclein oligomers and fibrils, disrupting their toxic effects and reducing neuronal damage. The study also hypothesized that NAbs within IVIg preparations could be used to specifically target disease-related aggregates in PD.

Rabenstein et al. (2019) also confirmed these data. They focused on the interaction between NAbs and toxic α-synuclein fragments, demonstrating that NAbs neutralized these fragments and reduced their cytotoxic effects. This highlights the role of NAbs in preventing aggregation and mitigating the downstream effects of α-synuclein toxicity.

Albus et al. (2018) expanded the understanding of NAbs functionality by demonstrating that NAbs against misfolded proteins, including α-synuclein, exhibit broad activity in clearing pathogenic aggregates in multiple neurodegenerative diseases. This study further suggested that NAbs present in IVIg could offer a multi-targeted approach for treating PD and other proteinopathies.

In conclusion, the presence of α-synuclein-specific NAbs in IVIg is effective in the treatment of PD by neutralizing toxic aggregates, inhibiting aggregation, and modulating neuroinflammation.

### 3.3 IVIg in autoimmune encephalitis

Autoimmune encephalitis (AE) is a severe and potentially life-threatening condition characterized by immune-mediated inflammation of the brain, often triggered by autoantibodies targeting neuronal surface antigens, synaptic proteins, or intracellular components. Several autoantibodies have been detected in AE, mostly directed against leucine-rich glioma inactivated protein 1 (LGI1), N-methyl-d-aspartate (NMDA) receptors, contactin-associated proteinlike 2 (CASPR2), and glutamic acid decarboxylase 65 (GAD65) (Irani, 2024).

IVIg is important in AE due to its ability to neutralize pathogenic autoantibodies, regulate immune responses, and promote neuronal survival. The effectiveness of IVIg in treating AE may be due to the presence of NAbs in the products. Dimitriadou et al. (2020) identified several anti-neuronal antibodies, including NAbs, in IVIg. These antibodies may neutralize pathogenic autoantibodies and modulate inflammation by suppressing complement activation and cytokine release. This dual action is particularly critical in AE, where neuronal damage and neuroinflammation coexist, exacerbating disease severity, thus, NAbs could be important in promoting the clearance of immune complexes and protecting neuronal integrity (Rodriguez et al., 2009). Moreover, IVIg influences immune cell behaviour by inhibiting T-cell proliferation, altering the Fc receptor expression on macrophages and microglia, and enhancing the activity of regulatory T cells. The differences in therapeutic responses to IVIg may be associated with the specific repertoire of anti-neuronal antibodies, which in turn affect the clinical outcomes (Dimitriadou et al., 2020).

From the clinical point of view, in a single-arm, open label trial, Lee et al. (2022) provided clinical data supporting the safety and efficacy of IVIg in AE. The study confirmed that IVIg led to significant clinical improvement in acute neurological symptoms. Yang and Liu (2021) demonstrated the efficacy of IVIg, particularly in refractory forms of AE. The review emphasized that the efficacy of IVIg derives from its ability to neutralize circulating autoantibodies, modulate the complement system, and regulate Fc receptor-mediated pathways (Yang and Liu, 2021). The benefit of IVIg in adult AE has been confirmed by a recent systematic review (Kaira et al., 2025).

Other studies reported the effectiveness of IVIg to treat AE in pediatric settings. Hill et al. (2023) conducted the IgNiTE trial, a randomized controlled study evaluating IVIg therapy in childhood encephalitis. The study demonstrated that IVIg significantly improved clinical outcomes, including neurological recovery and the reduction of long-term sequelae. This trial underscored the importance of IVIg in pediatric populations, where timely intervention can profoundly influence disease trajectory and neurodevelopmental outcomes.

Furthermore, the early use of IVIg is reported to be effective even in cases of AE triggered by infection (Karagianni et al., 2019). Infections can act as triggering factors through a mechanism of molecular mimicry, leading to the production of autoantibodies. IVIg is useful thanks to its immunomodulatory properties, capable of neutralizing autoantibodies and reducing inflammation, preventing further neuronal damage.

### 3.4 IVIg in small fiber neuropathy

Small fiber neuropathy (SFN) is a peripheral nerve disease that affects small sensory fibers (typically nociceptors), thinly myelinated Aδ fibers, and unmyelinated C fibers. It is characterized by severe neuropathic pain (Daifallah et al., 2023). The disease may be idiopathic (in 50% of cases) or caused by vitamin B12 deficiency, diabetes, and sodium channel gene mutations. It may also be associated with autoimmune conditions such as Sjogren’s syndrome, sarcoidosis, and celiac disease, in which autoantibodies target nervous system antigens. Symptoms are severe pain, tingling, orthostatic hypotension, dysautonomia, and sensory neuropathy (Gavrilova et al., 2022). SFN may be length-dependent, with a stocking-and-glove distribution. IVIg therapy and plasma exchange, which can block or remove freely circulating antibodies, can be correlated with relief of pain and other symptoms in SFN patients, thus suggesting the presence of pathogenic autoantibodies (Gavrilova et al., 2022). Autoantibodies have been identified in SFN that can bind small sensory neurons (Daifallah et al., 2023).

SFN is generally refractory to standard immunosuppressive therapies promoted for other systemic diseases. IVIg at a dose of 2 g/kg in 5 days and then 0.75–2 g/kg every 3–4 weeks may be beneficial in such patients, with a response in clinical and symptom severity (Gavrilova et al., 2022). NAbs present in IVIg may modulate the immune response by reducing inflammatory damage and exerting neuroprotective effects. However, the efficacy of IVIg therapy for SFN is different based on the underlying etiology. IVIg treatment has shown potential efficacy in SFN associated with celiac disease (Habek et al., 2011), but not in idiopathic or metabolic forms such as diabetes-related (Gibbons and Klein, 2021).

## 4 Therapeutic applications of NAbs in IVIg in autoimmune systemic and dermatological diseases

IVIg therapy is widely used in the treatment of autoimmune systemic and dermatological disorders (Table 3). These conditions are characterized by dysregulated immune responses, resulting in tissue damage, systemic inflammation, or immune-mediated cell apoptosis (Danieli et al., 2025).

**TABLE 3:** Natural autoantibodies present in intravenous immunoglobulin and their role in autoimmune systemic and dermatological diseases.

Publications	Type of study	Summary
Schluter et al. (2003)	Review in RA and SLE	NAbs against T-cell receptor (TCR) public idiotopes may downregulate autoreactive T-cell activity in immune-mediated diseases
György et al. (2008)	Research study in RA	NAbs targeting glycosaminoglycans have a role in modulating cytokine production and reducing synovial inflammation
Shoenfeld et al. (2002)	Preclinical study in SLE	IVIg-purified NAbs anti-dsDNA antibodies reduce lupus pathology, including proteinuria and kidney damage in a murine model
Su et al. (2008)	Comparative study in SLE	NAbs against phosphorylcholine in lupus patients have a protective role in apoptotic cell clearance and inflammation reduction
Luzi et al. (2009)	Review in SLE and ITP	Immunomodulatory effects of IVIg may be related to NAbs in maintaining immune homeostasis in SLE and ITP. (including complement inhibition, Fc receptor modulation, and increased regulatory T-cell function)
Burns and Franco (2015)	Review in KD	Immunomodulatory effects of IVIg in KD, including dampening of Toll-like receptor signaling, reduction of cytokine storms, and enhancing T regulatory cells
French et al. (2006)	Review in TEN	NAbs anti-Fas in IVIg inhibited *in vitro* and *in vivo* Fas-mediated keratinocyte apoptosis in TEN.
Ahmed et al. (2016)	Case series in PV	The combination therapy with IVIg and rituximab can be used successfully as first-line therapy in PV patients in whom systemic corticosteroids and immunosuppressants are contraindicated
Ahmed et al. (2024)	Review in PV	Outlined the role of IVIg in PV, emphasizing its capacity to neutralize pathogenic autoantibodies and restore immune tolerance, resulting in long-term remission

Abbreviations: IVIg, intravenous immunoglobulin; KD, kawasaki disease; NAbs, natural autoantibodies; PV, pemphigus vulgaris; RA, rheumatoid arthritis; SLE, systemic lupus erythematosus; TCR, T-cell receptor; TEN, toxic epidermal necrolysis.

### 4.1 Rheumatoid arthritis

RA is a chronic autoimmune disorder characterized by systemic inflammation, synovial hyperplasia, and progressive joint destruction. The disease is driven by autoantibodies, such as rheumatoid factor and anti-citrullinated protein antibodies, along with a dysregulated immune response involving T cells, B cells, and pro-inflammatory cytokines. IVIg therapy, containing NAbs, has shown promise in modulating immune responses and alleviating inflammation in RA.

György et al. (2008) identified NAbs reactive with glycosaminoglycans (GAGs) in RA patients. GAGs, such as hyaluronic acid and heparan sulphate, are essential components of the extracellular matrix in cartilage and synovial tissue. In RA, inflammatory processes lead to extracellular matrix degradation and exposure of GAGs, which become immunogenic. The study revealed that NAbs against GAGs are present in IVIg and may neutralize inflammatory responses triggered by GAG fragments. These NAbs modulate cytokine production and reduce synovial inflammation by targeting pro-inflammatory molecular patterns exposed in damaged tissuE.

Schluter et al. (2003) explored the role of NAbs targeting T-cell receptor (TCR) public idiotopes, which are conserved regions on TCRs shared across multiple T-cell clones. These NAbs are hypothesized to play a regulatory role in T-cell-mediated immunity. The authors claim NAbs against TCR public idiotopes may downregulate autoreactive T-cell activity, a critical driver of RA pathogenesis. By modulating TCR signalling, these NAbs contribute to immune tolerance and reduce synovial inflammation. Incorporation of these TCR-targeting antibodies in IVIg therapy provides an additional mechanism for controlling autoimmune T-cell responses in RA.

The insights provided by György et al. (2008) and Schluter et al. (2003) highlight opportunities to refine IVIg formulations, enhancing the concentrations of NAbs targeting GAGs and TCR idiotopes. Such targeted approaches could improve therapeutic outcomes while reducing the systemic burden of the disease.

### 4.2 Systemic lupus erythematosus

SLE is a complex autoimmune disease characterized by the production of a wide range of autoantibodies, including anti-dsDNA antibodies, which contribute to inflammation in multiple organs. IVIg therapy has therapeutic potential in SLE, with NAbs in IVIg playing critical immunoregulatory roles in the disease. Anti-idiotypic antibodies bind and neutralize pathogenic autoantibodies, such as anti-dsDNA. In a murine SLE model, Shoenfeld et al. (2002) demonstrated that IVIg reduced proteinuria, kidney damage, circulating immune complexes, and improved survival rates.

Another study investigated the potential of NAbs in SLE. Su et al. (2008) studied the role of NAbs against phosphorylcholine (PC) in SLE. Phosphorylcholine is a conserved epitope on apoptotic cells and oxidized lipids, frequently targeted by NAbs. The study documented lower levels of anti-PC antibodies in SLE patients compared to healthy controls, suggesting a protective role for these antibodies. Anti-PC NAbs in IVIg may enhance the clearance of apoptotic cells and reduce the inflammatory response triggered by the accumulation of cellular debris (Su et al., 2008).

Luzi et al. (2009) provided a comprehensive overview of the biological modulating molecules in IVIg, emphasizing its relevance in SLE. The authors described IVIg’s capacity to inhibit complement activation, preventing immune complex-mediated tissue damage. Moreover, IVIg enhances the activity of regulatory T-cell (Treg) and modulates the expression of Fc receptor on macrophages, thereby reducing their activation and the release of inflammatory cytokine (Luzi et al., 2009).

Finally, IVIg downregulates autoreactive B cells by binding to activating Fc receptors and modulating B-cell receptor signalling (Luzi et al., 2009), and suppresses pro-inflammatory cytokines such as IL-6, TNF-α, and IFN-α, mitigating the systemic inflammation characteristic of SLE.

NAbs within IVIg play a crucial role in modulating the immune dysregulation characteristic of SLE.

### 4.3 Kawasaki disease

Kawasaki disease (KD) is a systemic vasculitis primarily affecting children. The disease results from an exaggerated innate immune response triggered by environmental or infectious factors. IVIg is an effective therapy for KD, significantly reducing the risk of coronary artery aneurysms and improving clinical outcomes.

Burns and Franco (2015) reviewed the immunomodulatory actions of IVIg in Kawasaki disease (KD), highlighting the contribution of natural antibodies (NAbs). These antibodies modulate excessive activation of innate immune cells and inhibit TLR2/4 signaling, reducing pro-inflammatory cytokine release. They also regulate adaptive immunity by blocking autoreactive B cells through interaction with Fcγ receptors. Additional effects include inhibition of complement activation, promotion of apoptotic cell clearance, and suppression of matrix metalloproteinases (MMPs), all of which cumulatively limit endothelial injury, vascular remodeling, and aneurysm formation. These mechanisms underscore the central role of NAbs in IVIg efficacy and support efforts to optimize formulations and identify biomarkers of therapeutic response.

### 4.4 Immune thrombocytopenia

Immune thrombocytopenia (ITP) is an autoimmune disorder characterized by the destruction of platelets due to autoantibodies targeting platelet surface glycoproteins. This results in thrombocytopenia, leading to an increased risk of bleeding. IVIg is a first-line therapy for ITP, exerting its therapeutic effects through a combination of immunomodulatory mechanisms, including the activity of NAbs.

Luzi et al. (2009) reviewed the mechanisms by which IVIg treats ITP, including Fcγ receptor saturation, macrophage modulation, complement inhibition, and induction of regulatory T cells. Another mechanism is through anti-idiotypic antibodies. Anti-idiotypic NAbs in IVIg bind directly to pathogenic autoantibodies targeting platelet glycoproteins. By neutralizing these autoantibodies, IVIg reduces their ability to opsonize platelets and trigger their destruction.

IVIg has demonstrated consistent efficacy in increasing platelet counts and reducing bleeding risks in ITP patients. Its rapid onset of action (typically within 24–48 h) makes it an essential therapy in acute and severe ITP cases. Luzi et al. (2009) highlighted the role of NAbs in IVIg as key mediators of its therapeutic effects, emphasizing the importance of Fc receptor modulation and autoantibody neutralization.

### 4.5 Dermatological diseases

Dermatological conditions involving immune dysregulation, such as toxic epidermal necrolysis (TEN), Stevens-Johnson syndrome (SJS), and autoimmune blistering diseases, often require immunomodulatory therapies to control severe inflammation and tissue damage. IVIg, rich in NAbs, has demonstrated significant therapeutic potential in these conditions, primarily by modulating immune responses and attenuating inflammatory pathways. As in other autoimmune diseases, IVIg also modulates immune cells by reducing the production of pro-inflammatory cytokines, and suppressing complement activity (Danieli et al., 2025).

French et al. (2006) reviewed the role of IVIg in TEN and SJS. They noticed that IVIg inhibits keratinocyte apoptosis by blocking Fas-mediated signalling, reducing epidermal necrosis, and promoting re-epithelialization. In TEN, IVIg-derived NAbs interfere with Fas-FasL interactions, a key apoptotic pathway in keratinocyte death, thereby reducing epidermal detachment. The study highlighted the importance of early IVIg administration to maximize therapeutic benefits.

IVIg demonstrates efficacy even in other dermatological conditions such as pemphigus by neutralizing pathogenic autoantibodies, particularly through anti-idiotypic antibodies that mitigate tissue damage. Ahmed et al. (2016) proposed a first-line combined protocol based on IVIg and rituximab for patients with PV, bullous pemphigoid, mucous membrane pemphigus, ocular cicatricial pemphigoid, and epidermolysis bullosa acquisita (Ahmed Protocol). In this paper, the authors presented the data from seven published studies in patients with contraindications to systemic corticosteroids and immunosuppressive agents. The combination treatment with IVIg and rituximab decreased the skin inflammation in the microenvironment and promoted the restoration of immune homeostasis, leading to a sustained and prolonged disease and drug-free remission.

In their 20-year follow-up study of 21 patients, Ahmed et al. (2024) demonstrated that IVIg monotherapy was able to induce long-lasting clinical an serological remission, even in individuals refractory to conventional immunosuppressive treatments. The authors also highlighted the role of NAbs, abundantly present in IVIg preparations, which can regulate autoreactive clones and help maintain B and T cells homeostasis. These findings suggest that IVIg not only acts through its anti-inflammatory and immunomodulatory properties but may also contribute to the restoration of long-term immune tolerance in PV.

## 5 Discussion

Our review reported that the major evidence for the therapeutic benefit of NAbs in IVIg preparations concerns neurodegenerative diseases. The neuroprotective effect is linked to the presence of NAbs directed against specific elements implicated in pathological mechanisms, such as alpha-synuclein, tau protein, and amyloid-beta. In this way, NAbs promote the phagocytosis of Aβ aggregates and prevent the formation of Aβ oligomers and fibrils (Loeffler, 2023; Magga et al., 2010) and α-synuclein aggregates (Braczynski et al., 2022). Furthermore, specific NAbs, such as those neutralizing SAA, mediate an anti-neuroinflammatory action on microglia (Kuret et al., 2018). This anti-neuroinflammatory action is also important in other neurological pathologies, such as AE. In these pathologies, the use of IVIg has proven effective due to its anti-inflammatory effect and for the presence of antineuronal antibodies, which neutralize the pathological autoantibodies (Dimitriadou et al., 2020; Irani, 2024).

Evidence regarding the action of NAbs in specific autoimmune diseases is less consistent but encouraging. Given the importance of GAGs in promoting the inflammatory process underlying RA, it is evident that NAbs against GAGs play an active role in the therapeutic response. These antibodies neutralize GAG fragments, limiting the inflammatory response at the level of the synovial microenvironment (György et al., 2008). Neutralizing antibodies against TCR public idiotopes is also important; since they suppress the activity of autoreactive T cells (Schluter et al., 2003). Furthermore, in SLE anti-idiotypic NAbs bind and neutralize pathological autoantibodies such as anti-dsDNA or PC (Gigante et al., 2014). The presence of these specific NAbs in IVIg explains its efficacy in treating SLE. These NAbs promote the clearance of apoptotic cells, the reduction of the inflammatory response, and the activity of autoreactive B cells (Shoenfeld et al., 2002; Schluter et al., 2003; Luzi et al., 2009).

The presence of NAbs also justifies the efficacy of IVIg in other autoimmune diseases, such as ITP, KD, and some dermatological diseases. However, the literature provides insufficient evidence regarding specific mechanisms of action.

Therefore, from what has been reported, it is possible to identify specific NAbs contained in IVIg that can have an active therapeutic role in specific diseases. Identifying these NAbs is fundamental because it could lead to further therapeutic strategies. For example, one could think of IVIg formulations enriched with precise type of NAbs or pure NAbs concentrates for use in different clinical contexts.

The identification and characterization of specific NAbs found in healthy plasma donors would allow selective enrichment of IVIg preparations according to the targeted disease and render them more focused and less heterogeneous. In addition, purified NAbs can be formulated for direct use against specific pathologic targets, for example, oligomeric Aβ in Alzheimer’s disease or toxic α-synuclein in PD. Furthermore, the use of NAbs in treating autoimmune diseases has the advantage of reducing inflammatory responses without triggering the adverse effects characteristic of immunosuppressive therapies. The ability of certain NAbs to modulate inflammation, promote the removal of toxic antigens, or directly interact with key receptors (e.g., FcεRI on basophils) highlights the potential to isolate or engineer specific clones with therapeutic properties. This approach may also set the stage for the development of more specific therapies than conventional IVIg, therefore reducing variability in patient response and minimizing adverse effects.

## 6 Conclusion

NAbs play a crucial role in immune homeostasis, neuroprotection, and inflammation control, offering promising therapeutic potential in various diseases. Their presence in IVIg formulations has expanded immunoglobulin therapy, enabling targeted modulation of immune responses and protection against disease progression (Lobo, 2017; Kaveri, 2012; Fereidan-Esfahani et al., 2019; Schwartz-Albiez et al., 2009).

In neurodegenerative and autoimmune disorders such as Alzheimer’s, PD, SLE, and multiple sclerosis, NAbs help mitigate protein misfolding, neuroinflammation, and immune dysregulation (Kaveri and Bayry, 2017; Trépanier et al., 2012). Optimizing IVIg formulations to enhance these NAbs could improve therapeutic outcomes.

Future research should focus on refining IVIg preparations, enriching beneficial antibody subsets, and exploring their role in novel immunotherapies. Advanced translational research will further integrate NAbs into precision medicine, offering innovative strategies for managing autoimmune, inflammatory, and neurodegenerative diseases.

In conclusion, NAbs can potentially be used for developing novel personalized immunotherapies in the form of NAbs-rich preparations, specifically targeting the disease to be treated. The use of NAbs rather than routine IVIg application could facilitate the use of more specific therapy with fewer side effects, guiding clinical practice toward the precision immunology approach. Future research should target selective enrichment of therapeutic NAb subtypes in IVIg preparations, as well as the development of recombinant NAbs for use as precision therapeutics. This approach would take advantage of the intrinsic activities of NAbs, including the removal of apoptotic cells, neutralization of pathogenic autoantibodies, control of inflammation, and modulation of allergic responses, to generate new and more targeted treatments for autoimmune, inflammatory, neurological, and allergic disorders.
